# Survival outcomes of patients with muscle-invasive bladder cancer according to pathological response at radical cystectomy with or without neo-adjuvant chemotherapy: a case–control matching study

**DOI:** 10.1007/s11255-022-03339-6

**Published:** 2022-08-23

**Authors:** Noor van Ginkel, Tom J. N. Hermans, Dennie Meijer, Joost L. Boormans, Jens Voortman, Laura Mertens, Sytse C. van Beek, André N. Vis, the Dutch Cystectomy Snapshot Research Group, K. K. H. Aben, T. J. Arends, P. J. Ausems, D. Baselmans, C. P. A. M. Berger, A. C. Berrens, H. Bickerstaffe, S. D. Bos, M. Braam, K. T. Buddingh, S. Claus, K. Dekker, T. van Doeveren, S. M. H. Einerhand, L. M. C. L. Fossion, E. J. van Gennep, L. A. Grondhuis Palacios, F. J. Hinsenveld, M. M. Hobijn, S. H. van Huystee, M. Jaspers-Valentijn, O. S. Klaver, E. L. Koldewijn, L. Korsten, A. Lenting, K. J. Lentjes, H. B. Luiting, S. van der Meer, J. A. Nieuwenhuijzen, M. A. Noordzij, R. I. Nooter, C. A. W. Notenboom, R. J. A. Oomen, H. G. van der Poel, J. G. H. van Roermund, J. de Rooij, H. Roshani, D. K. E. van der Schoot, B. P. Schrier, M. A. van der Slot, D. M. Somford, P. J. Stelwagen, A. M. A. Stroux, A. van der West, B. P. Wijsman, W. A. K. M. Windt, P. van Zanten

**Affiliations:** 1 Department of Urology, Amsterdam University Medical Center, Location VUmc, De Boelelaan 1117, Postbus 7057, 1007 MB Amsterdam, The Netherlands; 2 Department of Urology, Maastricht University Medical Centre, Maastricht, The Netherlands; 3 Department of Urology, Erasmus University Medical Centre, Rotterdam, The Netherlands; 4 Department of Medical Oncology, Amsterdam UMC, Location VUmc, Amsterdam, The Netherlands; 5 Department of Urology, Netherlands Cancer Institute, Amsterdam, The Netherlands

**Keywords:** Chemotherapy, Muscle-invasive bladder cancer, Prognosis, Response, Prognosis

## Abstract

**Objectives:**

To assess survival of patients with muscle-invasive bladder cancer (MIBC) who underwent radical cystectomy (RC) with or without neo-adjuvant chemotherapy (NAC) according to the pathological response at RC.

**Methods:**

965 patients with MIBC (cT2-4aN0M0) who underwent RC with or without NAC were analyzed. Among the collected data were comorbidity, clinical and pathological tumor stage, tumor grade, nodal status (y)pN, and OS. Case–control matching of 412 patients was performed to compare oncological outcomes. Kaplan–Meier curves were created to estimate OS for patients who underwent RC with or without NAC, and for those with complete response (pCR), partial response (pPR), or residual or progressive disease (PD).

**Results:**

Patients with a pCR or pPR at RC, with or without NAC, had better OS than patients who had PD (both *p* values < 0.001). Moreover, the incidence of pCR was significantly higher in patients receiving NAC prior to RC than in patients undergoing RC only (31% versus 15%, respectively; *p* < 0.001). Case–control matching displayed better OS of patients who underwent RC with NAC, median survival not reached, than of those who underwent RC only, median 4.5 years (*p* = 0.023).

**Conclusions:**

This study showed that patients with MIBC who underwent NAC with RC had a significant better OS than those who underwent RC only. The proportion of patients with a pCR was higher in those who received NAC and RC than in those who were treated by RC only. The favorable OS rate in the NAC and RC cohort was probably attributed to the higher observed pCR rate.

**Supplementary Information:**

The online version contains supplementary material available at 10.1007/s11255-022-03339-6.

## Introduction

Patients with non-metastatic muscle-invasive bladder carcinoma (MIBC) have a 5-year overall survival (OS) rate of approximately 50% [1]. Cisplatin-based neo-adjuvant chemotherapy (NAC) prior to radical cystectomy (RC) leads to an OS benefit of 6–8% [1–3]. The greatest survival benefit of NAC is attributed to a subset of patients who experience a pathological complete response (pCR, ypT0N0) following NAC and RC [2, 3]. In patients who undergo RC without NAC, a pCR (i.e., stage pT0N0) is observed in 10–15% of the cases [4]. In non-randomized clinical trials, patients who experience a (y)pCR at RC with or without NAC showed 5-year survival rates of up to 75–85% [4, 5]. However, an exact comparison in OS between those who underwent NAC and RC, and RC only is difficult due to imbalances in prognostic factors. This underlines the current need for strategies to properly identify patients who are likely to benefit from NAC from those who do not. Since reliable clinical biomarkers for NAC response prediction are not available, proper evaluation of real-world data remains necessary. However, real-world studies reporting on the OS outcomes of patients with different pathological tumor stages (ypT) and with different nodal stages (ypN) after NAC are often limited by a relatively short follow-up time or residual confounding by imbalances in prognostic factors.

The primary aim of this study was to describe the intermediate-term OS of a large cohort of patients diagnosed with cT2-4aN0M0 urothelial MIBC treated by RC. The oncological outcome was compared for patients with a pathological complete response (pCR, ypT0N0), with a pathological partial (non-MIBC) response (pPR, (y)pT1/CIS N0), or with residual or progressive muscle-invasive disease (PD). Moreover, the OS of patients treated by means of RC only was compared with that of those treated by NAC and RC. To control for differences in pretreatment prognostic variables, a case–control matching study was performed to assess whether oncological outcomes remained.

## Methods

### Dutch cystectomy snapshot database

The Dutch Cystectomy Snapshot study is a nationwide retrospective multicenter observational cohort study reporting on the intermediate-term survival of open RC versus robot-assisted RC [6]. Details on the methods of this study have been described elsewhere [6]. In short, the study included 1609 patients with non-metastatic MIBC or high-risk non-MIBC who underwent RC between January 2012 and December 2015 as primary curative treatment (MEC-2018-1730, Erasmus Medical Center Rotterdam) in five university and 14 general hospitals in The Netherlands. Moreover, 19 of 47 (40%) hospitals in The Netherlands that perform RC participated in the Dutch Snapshot Cystectomy study. No selection based on hospital-volume or patient criteria was done for inclusion in the present study, thereby reflecting routine clinical care.

The following clinical data: age, gender, American Society of Anesthesiologists (ASA)-score, body mass index (BMI), Charlson comorbidity index (CCI), hemoglobin level, creatinine level at baseline, and tumor data: clinical staging according to the TNM classification, pathological tumor stage, and WHO-grade and follow-up data on clinical outcome were collected from the original patient charts. Any missing data on the clinical and pathological disease stage and the vital status of the patient were supplemented for completeness by data obtained from the Netherlands Cancer Registry (NCR), a nationwide network including all patients diagnosed with cancer based on the registry of histo- and cytopathology in The Netherlands (in Dutch: PALGA www.palga.nl).

### Patients in the present study: inclusion and exclusion criteria

For the present study, 982 patients with non-metastatic urothelial cell MIBC (i.e., cT2-4aN0M0) who underwent RC with or without NAC were analyzed. Patients were staged at diagnosis by computed tomography (CT) or position emission tomography (PET)/CT of thorax and abdomen, according to local hospital staging protocols. NAC consisted of combination therapy of Gemcitabine/Cisplatin, Gemcitabine/Carboplatin or Methotrexate, Vinblastine, Adriamycine, and Cisplatin. Patients who received experimental NAC combinations (*n* = 4) and patients with missing data on pathological disease stage at RC (*n* = 11) or type of NAC regimen (*n* = 2) were excluded. The decision to give NAC was based on local hospital protocol after a multidisciplinary tumor board meeting and therefore reflected real-world practice. Patients underwent either robot-assisted or open RC according to local clinical practice. Follow-up after surgery comprised of regular CT scanning of thorax and abdomen for all patients, and was in accordance with local hospital protocols compiled from international guidelines [4].

### Oncological outcome measures

The primary endpoint of the study was the OS, defined as the time from the date of RC until the date of death, from any cause. In case of unknown date of death, the patient was censored at the last follow-up date.

Survival data were stratified for pathological outcome at RC as a single modality treatment or NAC followed by RC. In this, pCR was defined as absence of tumor in the RC specimen and in the resected pelvic lymph nodes (y)pT0N0 or the presence of non-invasive papillary tumor (y)pTaN0. A pathological partial response (pPR) was defined as the presence of non-muscle-invasive bladder cancer and/or carcinoma in situ (CIS) in the RC specimen, (y)pT1/CIS N0. Residual or progressive disease (PD) at RC was defined as either residual organ-confined MIBC, (y)pT2N0, residual extra-vesical disease, (y)pT3-4N0, or residual or progressive node-positive disease, (y)pTanyN+ .

### Outcome variables and statistical analyses

Statistical analysis was performed using IBM^®^ SPSS^®^ Statistics version 25. Baseline characteristics and tumor variables were compared for those who underwent NAC and subsequent RC and those who underwent RC only using the Chi-square test (*χ*^2^) or Mann–Whitney *U* test (MWU) for categorical or continuous data, respectively (two-sided; *α* = 0.05). Survival data were compared for patients with pCR, pPR, or PD after RC, using Kaplan–Meier curves assessing OS. The log-rank test was used to compare survival estimates.

To correct for imbalances in prognostic factors at baseline, case–control matching was performed using the two cohorts (RC only = 0; NAC + RC = 1) as grouping variable. Age (categorical; < 60/60–74/ ≥ 75 years), gender (categorical; male/female), clinical tumor stage (categorical, ≤ cT2/ > cT2), Charlson comorbidity index (CCI) (categorical; 0–4/5–6/ > 6), and kidney function (categorical; creatinine < 100/ ≥ 100) were used as matching variables. Consequently, using the matched dataset, the incidence of pCR, pPR, and PD was compared for matched patients with and without NAC prior to RC using the Chi-square test. Subsequently, Kaplan–Meier curves were constructed that compared OS between patients who underwent NAC and RC with that of patients who underwent RC only.

To obtain predicted risks for patients with missing variables, a Bayesian stochastic regression imputation procedure was conducted. The imputation model consisted of the above listed variables in the models and the outcome variable (OS).

## Results

### Baseline characteristics of patients

In total, 965 patients with cT2-4aN0M0 MIBC were identified from the Dutch Cystectomy Snapshot database, of whom 739 (77%) received RC as a single treatment modality, and 226 (23%) were treated with NAC followed by RC. Table 1 shows the baseline characteristics of the patient population. Patients who received NAC and RC were younger (median 65, IQR 59–71) than those who underwent RC only (median 69, IQR 63–75), had less comorbidities (CCI 0–4: 81% versus 66%), and had more advanced clinical tumor stage (> cT2: 57% versus 24%). The median follow-up time of patients who received NAC and RC was 3.4 years (IQR 1.6–5.1), compared to 3.5 years (IQR 1.1–5.0) in patients who underwent RC only (*p* = 0.17).

**Table 1:** Baseline characteristics and pathological outcomes of 965 patients with muscle-invasive bladder cancer (MIBC) who were treated by radical cystectomy (RC) only versus patients who were treated by neo-adjuvant chemotherapy (NAC) followed by RC

	All patients (*n* = 965)	RC only (*n* = 739)	NAC + RC (*n* = 226)	*p* value
Age at surgery, years; median (IQR)	68 (62–74)	69 (63–75)	65 (59–71)	< 0.001
Pre-operative creatinine level, μmol/L; median (IQR)	89 (75–106)	88 (74–103)	96 (78–112)	< 0.001
Pre-operative hemoglobin level, mmol/L; median (IQR)	8.2 (7.3–9.0)	8.5 (7.6–9.1)	7.2 (6.6–7.9)	< 0.001
Body mass index, kg/m^2^; median (IQR)	25 (23–28)	26 (23–28)	25 (23–28)	0.38
Gender; *n* (%)				
Male	704 (73)	540 (73)	164 (73)	0.88
Female	261 (27)	199 (27)	62 (27)	
Clinical T-stage; *n* (%)				
cT2	657 (68)	561 (76)	96 (43)	< 0.001
cT3	264 (27)	153 (21)	111 (49)	
cT4a	44 (5)	25 (3)	19 (8)	
ASA-score; *n* (%)				
I	161 (17)	127 (17)	34 (15)	0.32
II	557 (58)	411 (56)	146 (64)	
III	169 (17)	129 (17)	40 (18)	
IV	5 (< 1)	5 (1)	0 (0)	
Missing	73 (8)	67 (9)	6 (3)	
Charlson comorbidity index; *n* (%)				
0–4	670 (70)	486 (66)	184 (81)	< 0.001
5–6	205 (21)	170 (23)	35 (16)	
> 6	80 (8)	74 (10)	6 (3)	
Missing	10 (1)	9 (1)	1 (< 1)	
NAC regimen; *n* (%)				
Gem/Cis	162 (71)	–	162 (71)	–
Gem/Carbo	35 (16)	–	35 (16)	
MVAC	29 (13)	–	29 (13)	
Number of NAC cycles; *n* (%)				
1–2	15 (7)	–	15 (7)	**–**
3	57 (27)	–	57 (27)	
≥ 4	138 (66)	–	138 (66)	
Pathological T-stage; *n* (%)				
pT0	163 (17)	89 (12)	74 (33)	< 0.001
pTa	8 (1)	7 (1)	1 (< 1)	
CIS	48 (5)	34 (4)	14 (6)	
pT1	44 (4)	27 (4)	17 (8)	
pT2	232 (24)	192 (26)	40 (18)	
pT3	356 (37)	294 (40)	62 (27)	
pT4	114 (12)	96 (13)	18 (8)	
Pathological N-stage; *n* (%)				
pN0	725 (75)	543 (73)	182 (80)	0.16
pN1	118 (12)	96 (13)	22 (10)	
pN2	119 (12)	97 (13)	22 (10)	
pN3	3 (1)	3 (1)	0 (0)	
Number of removed lymph nodes; median (IQR)	15 (10–20)	15 (10–19)	17 (11–23)	< 0.001
Surgical margin status; *n* (%)				
Negative	848 (88)	641 (87)	207 (92)	0.035
Positive	95 (10)	81 (11)	14 (6)	
Missing	22 (2)	17 (2)	5 (2)	
Pathological response; *n* (%)				
Complete response (pCR)	161 (17)	91 (12)	70 (31)	< 0.001
Partial response (pPR)	87 (9)	57 (8)	30 (13)	
Progressive disease (PD)	717 (74)	591 (80)	126 (56)	

*RC* radical cystectomy, *NAC* neo-adjuvant chemotherapy, *IQR* interquartile range, *ASA* American Society of Anesthesiologists, *Gem/Cis* gemcitabine/cisplatin, *Gem/Carbo* gemcitabine/carboplatin, *MVAC* methotrexate, vinblastine, adriamycine, and cisplatin

### Pathological outcomes in the RC specimen

Table 1 shows the pathological outcomes in the RC specimen of patients treated with RC only and patients who underwent NAC followed by RC. The rates of ypCR, ypPR, and PD in patients treated with NAC were 31% [70/226, 95% confidence interval (CI) 25–37%], 13% (30/226, 95% CI 9–18%), and 56% (126/226, 95% CI 49–62%), respectively. For patients who received RC, the rates of pCR, pPR, and PD were 12% (91/739, 95% CI 10–15%), 8% (57/739, 95% CI 6–10%), and 80% (591/739, 95% CI 77–83%), respectively.

### Survival outcomes after RC stratified per pathological outcome

**Fig. 1:**
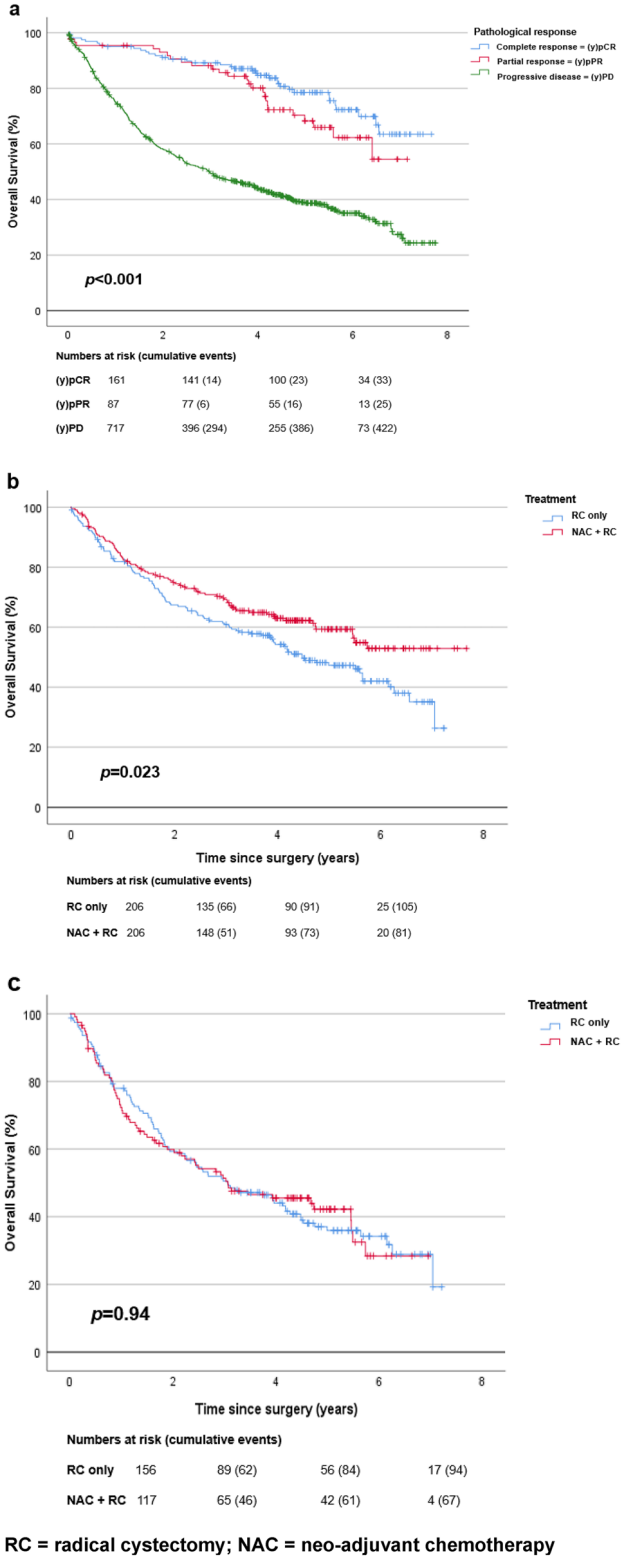
Kaplan Meier curves. **a** Overall survival in 965 patients with muscle-invasive bladder cancer who were treated by radical cystectomy (RC) with or without neo-adjuvant chemotherapy (NAC), stratified by pathological response at RC. **b** Overall survival after case–control matching of patients with muscle-invasive bladder cancer treated with NAC followed by RC (*n* = 206, curve in red) versus patients treated by RC only (*n* = 206, curve in blue). **c** Overall survival after case–control matching of patients with residual muscle-invasive disease at radical cystectomy (RC) for patients treated by RC only (*n* = 156, curve in blue) and those treated with NAC followed by RC (*n* = 117, curve in red)

Figure 1a shows the estimates of OS of patients treated with and without NAC, stratified by pathological response at RC. For patients with pCR and pPR, the median OS were not reached, while patients with PD had a median OS of 3.0 years. For patients with pCR, pPR, and PD, the five-year survival rates were 82% (95% CI 76–88%), 72% (95% CI 63–82%), and 40% (95% CI 36–43%), respectively. Patients with pCR or pPR had a significantly better OS than patients with PD at RC (*p* < 0.001).

For patients who underwent RC only with pCR, pPR, and PD, the 5-year survival rates were 77% (95% CI 68–86%), 74% (95% CI 62–85%), and 40% (95% CI 36–44%). In patients who underwent NAC and RC with pCR, pPR, and PD, the 5-year survival rates were 89% (95% CI 81–97%), 70% (95% CI 53–87%), and 40% (95% CI 32–49%), respectively.

### Case–control matching study

After case–control matching, 412 patients (206 patient per cohort) were matched in both cohorts. Table 2 shows baseline characteristics of the patients who underwent RC and those who underwent NAC followed by RC after case–control matching. The preoperative hemoglobin level differed significantly between both cohorts (median 8.4 mmol/l in the cohort treated by RC only, median 7.2 mmol/l in the cohort treated by NAC followed by RC; *p* < 0.001). No differences were found in BMI (*p* = 0.71), presence of CIS (*p* = 0.66), and ASA-score (*p* = 0.19). As the remaining variables were used as matching variables, no differences were noted between both cohorts *(p* = 1.0).

**Table 2:** Baseline characteristics and pathological response of 412 patients after case–control matching with cT2-4N0M0 muscle-invasive bladder cancer (MIBC) who were treated by either radical cystectomy as a single treatment modality versus patients who underwent neo-adjuvant chemotherapy (NAC) followed by radical cystectomy (RC)

	All patients (*n* = 412)	RC only (*n* = 206)	NAC + RC (*n* = 206)	*p* value
Age at surgery, years; *n* (%)				
< 60	170 (41)	85 (41)	85 (41)	1.0
60–74	222 (54)	111 (54)	111 (54)	
≥ 75	20 (5)	10 (5)	10 (5)	
Pre-operative creatinine level, μmol/L; *n* (%)				
< 100	248 (60)	124 (60)	124 (60)	1.0
≥ 100	164 (40)	82 (40)	82 (40)	
Gender; *n* (%)				
Male	298 (72)	149 (72)	149 (72)	1.0
Female	114 (28)	57 (28)	57 (28)	
Clinical T-stage; *n* (%)				
cT2	192 (47)	96 (47)	96 (47)	1.0
cT3-4a	220 (53)	110 (53)	110 (53)	
Charlson comorbidity index; *n* (%)				
0–4	330 (80)	165 (80)	165 (80)	1.0
5–6	70 (17)	25 (17)	25 (17)	
> 6	12 (3)	6 (3)	6 (3)	
Pathological response; *n* (%)				
Complete response (pCR)				
No	317 (77)	176 (85)	141 (68)	< 0.001
Yes	95 (23)	30 (15)	65 (32)	
Partial response (pPR)				
No	368 (89)	186 (90)	182 (88)	0.52
Yes	44 (11)	20 (10)	24 (12)	
Progressive disease (PD)				
No	139 (34)	50 (24)	89 (43)	< 0.001
Yes	273 (66)	156 (76)	117 (57)	

*RC* radical cystectomy, *NAC* neo-adjuvant chemotherapy, *IQR* interquartile range, *ASA* American Society of Anesthesiologists

Figure 1b shows the estimates of OS for case–control-matched patients who underwent RC as a single modality and those who underwent NAC followed by RC. A statistically significant difference was found regarding OS (*p* = 0.023) between both cohorts. For patients who underwent RC only, the median OS was 4.5 years. In patients who underwent NAC and RC, the median OS was not reached. For patients who underwent RC only, the 5-year survival rate was 49%, and for patients who underwent NAC followed by RC, the 5-year survival rate was 60%.

In Table 2, the incidence of patients who had pCR, pPR, and PD is reported for both matched cohorts. A statistically significant difference was found for pathological outcome after RC, with a higher proportion of patients who had pCR in the cohort of patients in the NAC plus RC treatment regimen than in the RC only group (32% versus 15%, *p* < 0.001). These figures were 12% versus 10% (*p* = 0.52) for those with pPR, and 57% versus 76% (*p* < 0.001) for those with PD, respectively.

Figure 1c depicts the OS for case–control-matched patients with yPD versus those with PD. No differences were observed between both patient cohorts (*p* = 0.94).

## Discussion

In this multicenter, population-based study of 965 patients with cT2-4aN0M0 MIBC, we assessed the oncological outcome of patients with MIBC who underwent RC with or without NAC. Besides, we aimed to compare the oncological outcome in patients with a pathological complete response (pCR), a pathological partial response (pPR), or with progressive disease (PD) after RC. We found that patients without residual muscle-invasive disease (pCR or pPR) had a better intermediate-term OS than patients with residual muscle-invasive disease. The number of patients with pCR was significantly higher in patients treated with NAC compared to RC only. After case–control matching for several known prognostic variables, NAC prior to RC was significantly associated with improved OS. It is assumable that the observed improved survival can be attributed to this higher rate of pCR disease in the NAC cohort.

The pCR rate in the NAC and RC group of 31% (70/226) is comparable to what has been reported in previous randomized clinical trials (RCTs). In the landmark SWOG study, 38% of patients receiving NAC had a pCR at RC [4]. In a meta-analysis of 1734 patients from 12 RCTs, approximately 25% of patients had ypT0 after NAC [6]. However, two retrospective multicenter series reported ypCR rates of only 13 and 23% indicating that a substantial difference to RCT cohorts was observed [3, 7]. Since a ypCR following NAC has been strongly associated with an OS benefit, the differences in the rate of pathological outcome after RC in observational studies raise concerns regarding the true effectiveness of NAC in real-life world [2, 8]. The observed difference with other retrospective multicenter series is possibly due to lower inclusion rates of clinically T4a tumors in our study, i.e., only 8% in our study versus 15% in the group of Zargar et al. in whom lower pCR rates were observed.

Our results are in accordance with previous retrospective studies and RCTs which reported a 5-year OS of patients with a ypCR after NAC ranging from 80 to 89% [4, 8–10]. In a recent study, the 5-year risk of recurrence in patients with ypT0-disease was only 9% (95% CI 2–12). Unfortunately, the follow-up in most ‘real-world’ cohorts in which oncological outcomes of different pathological stages were analyzed after NAC, were relatively short, i.e., 1.4 to 2.6 years [9, 10] or subject to ‘residual confounding’ [5]. This leads to limitations in analyses on OS of more favorable prognostic patient groups. With a sufficient intermediate- to long-term median follow-up of 4.2 years, our study is able to re-affirm the excellent survival outcomes of patients with a pCR after NAC and RC in a ‘real-world’ setting, i.e., a 5-year OS of 89% (95% CI 81–97%).

Because of the advantageous prognosis for those with a pCR, there is an increasing interest in the search for preoperative identifiers to predict a complete response to NAC. In fact, the excellent survival rates of patients with pCR contribute to the debate whether they would benefit from RC or whether they could potentially be treated with a bladder-sparing protocol. It was also as suggested recently that a less stringent follow-up regimen could be applied in those with pCR patients after RC [10]. To date, however, it is not yet possible to accurately identify patients with a pCR after NAC [11]. In the present study, a logistic regression model showed no significant association of clinical parameters age, gender, ASA-score, CCI, preoperative creatinine, clinical tumor stage, type of NAC regimen, and completion of NAC cycles with pCR (online supplemental Table 1). In previous studies, factors that may influence the rate of downstaging on NAC included (lower) original stage, tumor size, and complete transurethral resection [3, 12–14]. In the past few years, several biomarker studies focused on genetic tumor characteristics which could help in further tailoring bladder-cancer care. Studies in small patient cohorts reported mutations in the DNA damage response genes such as ATM, RB1, FANCC, ERBB2, and ERCC2 to be enriched in patients with MIBC who experienced a pCR after NAC, or mismatch repair genes *MSH2* and *MLH1* which contribute to cisplatin resistance [15–18]. Retrospective studies showed a different clinical behavior in those with different molecular subtypes [19]. As caution must be taken when active surveillance is considered [11], prospective clinical trials for response prediction and evaluation are being conducted; for example, the PRE-PREVENCYS study in which biomarker analyses of blood, urine, and tissue and a re-staging TUR are combined to predict pCR at RC [20]**.**

Some other interesting observations were done in the present cohort. In contrast to the excellent survival of pCR patients, there is accumulating evidence for an adverse oncological outcome of patients with residual MIBC after NAC and RC, compared to patients with the same pathological disease stage after RC alone. In our case–control matching study, we did not observe a difference in intermediate-term OS between patients with yPD in comparison with PD (*p* = 0.94). Apparently, NAC does not seem to impair the prognosis of patients with PD after RC despite individual cases in which tumors seem to be resistant to chemotherapy. There was a significant worse 5-year OS for those experiencing residual ypT3-4N0 or ypTanyN+ disease than those with residual ypT2N0 disease after NAC and RC (*p* < 0.001, data not shown). These findings suggest that, patients with residual MIBC after NAC, specifically those with residual high-risk ypT3-4N0 and ypTanyN + disease, might benefit from adjuvant therapies such as immunotherapy [21].

The present study is limited by its observational and retrospective nature which might have led to an imbalance in prognostic factors, even after case–control matching. The preoperative hemoglobin and creatinine were available, while these might be influenced by NAC. Unfortunately, data on ‘high–risk’ and ‘very high risk NMIBC’ were not available. It is assumed that these patients did not undergo NAC before radical surgery. Possibly, clinical tumor stadium did not sufficiently represent tumor load and the improved survival after NAC is explained by differences in case-mix (Fig. 1b). Not all data relevant to the decision to give NAC were collected such as presence of hydronephrosis on dissemination imaging studies or staging results from magnetic resonance imaging (MRI), making definite comparisons difficult. Finally, data on adjuvant therapy on tumor recurrence were not retrieved.

High accuracy of all collected data, in particular the primary endpoint, was accomplished by supplementing data from the database of the Dutch Association of Urology with data from the local patient report, the NCR database and municipal registers in the NCR database. These data collection efforts resulted in sufficient follow-up time and complete data on disease recurrence. This is in contrast to previous real-world studies reporting on OS outcomes as they are often limited by a relatively short follow-up time or residual confounding by imbalances in prognostic factors.

With the inclusion at least half of all eligible Dutch patients with MIBC treated between 2012 and 2015, the present study is an adequate reflection of the use and effectiveness of NAC in recent routine clinical practice in The Netherlands. No selection of hospitals based on volume was done, resulting in an inclusion of 14 general and 5 academic hospitals. This is where the value of this article lies: the consolidation of the value of NAC in daily clinical practice, both in high-volume academic hospitals as in regional hospitals. This evaluation of real-world data is necessary because of possible selection bias in RCTs and the risk of limited external validity.

## Conclusion

The present study showed that patients with MIBC who underwent NAC and subsequent RC had a significant better OS than those who underwent RC as a single treatment modality. This improved oncological outcome remained after case–control matching for differences in patient selection. The frequency of pCR was statistically significantly higher in those who received NAC and RC than those with RC only (31% vs. 12%), leading to an excellent 5-year OS of 89% (95% CI 81–97%). The improved OS rate in the NAC and RC cohort was probably attributed to the higher observed pCR rate. For patients with MIBC, as long as accurate response prediction or evaluation is still not possible, these results emphasize the need for administration of NAC before RC if possible.

## Supplementary Information

Below is the link to the electronic supplementary material.

Supplementary file1 (DOCX 553 KB)
