# Supplementary material for: Survival outcomes of patients with muscle-invasive bladder cancer according to pathological response at radical cystectomy with or without neo-adjuvant chemotherapy: a case–control matching study
Source: Int Urol Nephrol. 2022 Aug 23;54(12):3145–52. doi: 10.1007/s11255-022-03339-6 (PMC9606088; doi:10.1007/s11255-022-03339-6)
Supplement: Supplementary file 1 — Supplementary file1 (DOCX 553 KB) [file 11255_2022_3339_MOESM1_ESM.docx]

**Online supplemental table 1** – Multivariable logistic regression analysis of factors associated with pathological complete downstaging (ypT0/aN0) in patients with cT2-4aN0M0 muscle-invasive bladder cancer treated by neoadjuvant chemotherapy and radical cystectomy (n=226).

|  |  | Complete pathological downstaging (ypT0/aN0)^a^ | | | | | | | | |
| --- | --- | --- | --- | --- | --- | --- | --- | --- | --- | --- |
|  |  | **Univariable logistic regression** | | |  | | **Multivariable logistic regression** | | | |
|  |  | OR | 95% CI | p-value | |  | | OR | 95% CI | p-value |
| Age, per 10 years |  | 1.21 | 0.88-1.66 | 0.25 | |  | | 1.22 | 0.81-1.26 | 0.34 |
| Female sex |  | 0.71 | 0.37-1.37 | 0.30 | |  | | 0.72 | 0.37-1.41 | 0.34 |
| ASA score |  | 1.15 | 0.70-1.89 | 0.59 | |  | |  |  |  |
| I |  | reference |  |  | |  | |  |  |  |
| II |  | 0.77 | 0.35-1.70 | 0.51 | |  | |  |  |  |
| III |  | 1.11 | 0.43-2.90 | 0.82 | |  | |  |  |  |
| CCI |  | 1.08 | 0.91-1.27 | 0.39 | |  | | 1.01 | 0.81-1.26 | 0.91 |
| Clinical T stage |  |  |  |  | |  | |  |  |  |
| cT2 |  | reference |  |  | |  | | reference |  |  |
| cT3 |  | 1.26 | 0.70-2.28 | 0.44 | |  | | 1.30 | 0.72-2.37 | 0.39 |
| cT4 |  | 0.65 | 0.20-2.12 | 0.47 | |  | | 0.61 | 0.19-2.03 | 0.42 |
| Type of NAC |  |  |  |  | |  | |  |  |  |
| Gem/Cis |  | reference |  |  | |  | |  |  |  |
| Gem/Carbo |  | 0.75 | 0.33-1.72 | 0.50 | |  | |  |  |  |
| MVAC |  | 1.15 | 0.50-2.64 | 0.75 | |  | |  |  |  |
| NAC all cycles completed |  |  |  |  | |  | |  |  |  |
| Yes |  | reference |  |  | |  | | reference |  |  |
| No |  | 0.75 | 0.40-1.40 | 0.36 | |  | | 0.75 | 0.39-1.45 | 0.39 |
| Abbreviations: CI, confidence interval; Gem/Carbo, gemcitabine/carboplatin; Gem/Cis, Gemcitabine/Cisplatin; MVAC, methotrexaat, vinblastine, adriamycine and cisplatin; NAC, neoadjuvant chemotherapy; OR, odds ratio  The model included 226 patients and 70 events. | | | | | | | | | | |
